# The role of the brainstem in sleep disturbances and chronic pain of Gulf War and Iraq/Afghanistan veterans

**DOI:** 10.3389/fnmol.2023.1266408

**Published:** 2024-01-08

**Authors:** Yu Zhang, Matthew Moore, Jennifer S. Jennings, J. David Clark, Peter J. Bayley, J. Wesson Ashford, Ansgar J. Furst

**Affiliations:** ^1^War Related Illness and Injury Study Center, VA Palo Alto Health Care System, Palo Alto, CA, United States; ^2^Department of Psychiatry and Behavioral Sciences, Stanford University School of Medicine, Stanford, CA, United States; ^3^Anesthesiology Service, VA Palo Alto Health Care System, Palo Alto, CA, United States; ^4^Department of Anesthesiology, Perioperative and Pain Medicine, Stanford University School of Medicine, Stanford, CA, United States; ^5^Department of Neurology and Neurological Sciences, Stanford University School of Medicine, Stanford, CA, United States

**Keywords:** chronic multisymptom illness (CMI), Gulf War, Iraq and Afghanistan Wars, sleep difficulty, chronic pain, magnetic resonance imaging (MRI), diffusion tensor image (DTI), brainstem

## Abstract

**Introduction:**

Gulf War Illness is a type of chronic multisymptom illness, that affects about 30% of veterans deployed to the 1990–91 Persian Gulf War. Veterans deployed to Iraq/Afghanistan after 2000 are reported to have a similar prevalence of chronic multisymptom illness. More than 30 years after the Persian Gulf War, Gulf War Illness still has an unexplained symptom complex, unknown etiology and lacks definitive diagnostic criteria and effective treatments. Our recent studies have found that substantially smaller brainstem volumes and lower fiber integrity are associated with increased sleep difficulty and pain intensity in 1990–91 Persian Gulf War veterans. This study was conducted to investigate whether veterans deployed to Iraq/Afghanistan present similar brainstem damage, and whether such brainstem structural differences are associated with major symptoms as in Gulf War Illness.

**Methods:**

Here, we used structural magnetic resonance imaging and diffusion tensor imaging to measure the volumes of subcortices, brainstem subregions and white matter integrity of brainstem fiber tracts in 188 veterans including 98 Persian Gulf War veterans and 90 Iraq/Afghanistan veterans.

**Results:**

We found that compared to healthy controls, veterans of both campaigns presented with substantially smaller volumes in brainstem subregions, accompanied by greater periaqueductal gray matter volumes. We also found that all veterans had reduced integrity in the brainstem-spinal cord tracts and the brainstem-subcortical tracts. In veterans deployed during the 1990–91 Persian Gulf War, we found that brainstem structural deficits significantly correlated with increased sleep difficulties and pain intensities, but in veterans deployed to Iraq/Afghanistan, no such effect was observed.

**Discussion:**

These structural differences in the brainstem neurons and tracts may reflect autonomic dysregulation corresponding to the symptom constellation, which is characteristic of Gulf War Illness. Understanding these neuroimaging and neuropathological relationships in Gulf War and Iraq/Afghanistan veterans may improve clinical management and treatment strategies for modern war related chronic multisymptom illness.

## 1 Introduction

About 30% of the veterans who deployed to the August 1990–May 1991 Persian Gulf War have developed a constellation of chronic symptoms (Kang et al., 2000; Smith et al., 2013). The chronic multisymptom illness (CMI) now referred to as Gulf War Illness (GWI) was first described in Air Force veterans involved in Operation Desert Shield/Desert Storm (ODS/DS) (Fukuda et al., 1998) as one or more unexplained symptoms, that present for 6 months or longer, in at least two of the three symptom categories: (1) fatigue and sleep difficulties, (2) musculoskeletal pain, and (3) mood and cognitive symptoms as well as neurological conditions. These widespread multi-symptom patterns are common in modern wars (Walker et al., 2010). In particular, some studies (Gironda et al., 2006; McAndrew et al., 2016a) reported veterans who served in Iraq and/or Afghanistan [Operation Enduring Freedom/Operation Iraqi Freedom (OEF/OIF)] from September 11, 2001 to now present with multiple chronic symptoms that appear to have similarities with GWI. However, there might also be differences in symptoms between the older ODS/DS vs. younger OEF/OIF cohorts. For example, a Millennium study reported that OEF/OIF veterans have higher prevalence of chronic pain and anxiety than those deployed to ODS/DS (Smith et al., 2014). Likewise, those deployed to ODS/DS have a higher prevalence of fatigue and memory problems than OEF/OIF cohorts. This may partly be due to ODS/DS veterans also being substantially older than OIF/OEF veterans. Another study found 1 year after deployment, 50% of OEF/OIF veterans met mild to moderate CMI, and 10% met severe CMI (McAndrew et al., 2016b). One important consideration of the causes of CMI is that there are many environmental exposures that lead combat veterans to have a higher incidence of a particular variety of symptoms. The environmental exposures in these Middle East Wars, including sand dust, oil well fires, burn pits, chemical weapons, chemical nerve agent antidotes [pyridostigmine bromide (PB) or nerve agent pyridostigmine pretreatment (NAPP)], some vaccines not previously used by the military, depleted uranium munitions, as well as hearing chemical alarms or wearing MOPP gear, have been suggested to be related to CMI. The major symptoms of CMI, including sleep problems and pain, have been hypothesized to be associated with dysregulation of the autonomic system or the hypothalamic-pituitary-adrenal (HPA) axis. Research suggests that some symptoms, such as chronic fatigue, may be due to subtle autonomic system dysfunction (Haley et al., 2013; Rayhan et al., 2013). Studies also reported neuroendocrine dysregulation of the HPA axis in Gulf War veterans in response to fibromyalgia (Buskila, 2000) and stress-related conditions such as post-traumatic stress disorder (PTSD) (Golier et al., 2006, 2007). Both the autonomic system and HPA are linked to central neural circuitry in structures of the brainstem (Ulrich-Lai and Herman, 2009).

Magnetic resonance imaging (MRI) has been used to identify structural and functional changes in the brain of veterans with GWI (Rayhan et al., 2013). Some previous studies have highlighted abnormal brainstem findings in GWI. For example, smaller brainstem volume has been found in Gulf War veterans using this technique (Christova et al., 2017; Zhang et al., 2020a). One study reported that veterans with GWI, relative to patients with Myalgic Encephalomyelitis and Chronic Fatigue Syndrome, showed functional deactivation within dorsal midbrain and cerebellar vermis in response to exercise (Washington et al., 2020). Another study reported that GWI with exercise induced orthostatic tachycardia is associated with brainstem atrophy (Rayhan et al., 2013). One of our recent studies reported that in 90 GWI veterans, poorer sleep quality correlated with smaller brainstem volume and lower fractional anisotropy (FA) of fiber tracts connecting the brainstem to basial ganglia and prefrontal cortices. Greater pain intensity correlated with reduced integrity of fiber tracts connecting the brainstem to the hypothalamus (Zhang et al., 2021).

Previous brain imaging studies have focused on diagnosis and understanding of GWI in veterans from the 1990–91 conflicts. However, information regarding CMI in OEF/OIF veterans is very limited. With a high prevalence of CMI in OEF/OIF veterans, there is an urgent need to determine whether brainstem structural and functional abnormalities are also found in this population. The major goals of this study were (1) to test whether ODS/DS and OEF/OIF veterans present brainstem abnormalities when compared to healthy control data from publicly available multi-center cohorts; (2) whether OEF/OIF veterans present similar brainstem correlations with sleep and pain severity as we previously observed in ODS/DS veterans. As previous research has suggested that the periaqueductal gray (PAG), the locus coeruleus (LC) and the rostral ventromedial medulla (RVM) are key nuclei participating in descending pain modulation functions, this study aimed to explore the relationship between pain and volumetric measures of the PAG, LC and RVM. (3) Lastly, the study tested whether the relationships of the major symptoms in CMI and brain morphometry/function are different between veterans of the two campaigns.

## 2 Materials and methods

### 2.1 Subjects

All veteran data were collected at the War Related Illness and Injury Study Center (WRIISC) at the Veterans Affairs (VA) Hospital in Palo Alto, CA, USA. The study protocol was approved by the Stanford University Institutional Review Board. All veterans provided written informed consent to participate in the study and shared their data in accordance with the Declaration of Helsinki. Inclusion criteria included: (1) veterans who were deployed to combats in the Middle East, either during ODS/DS between August 1990 and May 1991 Persian Gulf War or to OEF/OIF in Iraq/Afghanistan between 2001 through present; (2) had completed MRI scans, self-report questionnaires, and clinical reports including diagnosis and screening, neuropsychological assessments by a WRIISC psychologist, neurologist, and psychiatrist. Exclusion criteria included: (1) poor MRI quality; (2) incomplete, or insufficient sleep or pain evaluations; or (3) serious neurological or neuropsychiatric conditions with known etiology e.g., multiple sclerosis, Parkinson’s disease, epilepsy, stroke, dementia, schizophrenia, or prior neurosurgical interventions in the brain.

To replicate our previous findings of smaller brainstem volume in Gulf War (i.e., ODS/DS) veterans (Zhang et al., 2020a), we used new imaging data including 96 age- and gender-matched healthy controls (HC) from public data sources of two study cohorts. Of the total 96 HC, 56 were obtained from the National Institute of Mental Health (NIMH) Data Archive^1^ (Nugent et al., 2022), and 40 were collected from the Image and Data Archive^2^ of the Parkinson’s Progression Marker Initiative (PPMI) (Parkinson Progression Marker Initiative, 2011). Notably, there was no overlap of the PPMI data in this study with the PPMI data we used in our previous study (Zhang et al., 2020a). The detailed demographic information, MRI site, scanner, and parameter features are listed in Table 1.

**TABLE 1:** Details of the entire data collected across study cohorts.

Cohort	NIMH	PPMI	WRIISC-CA
No. of subjects	56	40	188
Age (mean ± SD)	38.6 ± 12	55.7 ± 9.6	45.1 ± 9.4
Age range (years)	24–71	31–70	23–69
Sex No. of men (%)	40 (71%)	37 (93%)	168 (89%)
Years of education (mean ± SD)	–	15.4 ± 2.8	14.4 ± 2.2
Education levels* (mean ± SD)	3.70 ± 0.57	2.30 ± 1.45	**1.63 ± 1.44**
Handedness No. of right (%)	44 (79%)	31 (78%)	151 (80%)
Ethnicity No. of White (%)	40 (71%)	35 (88%)	145 (77%)
Clinical diagnosis	Healthy control	Healthy control	Chronic Multisymptom Illness
No. of site	1	9	1
MR scanner	GE Discovery MR750	(1) Siemens TrioTim, (2) Siemens Verio	GE Discovery MR750
Field strength	3 Tesla	3 Tesla	3 Tesla
T1WI acquisition	Axial 3D-MPRAGE	Sagittal 3D-MPRAGE	Sagittal 3D-FSPGR
T1WI voxel size (mm)	1 × 1 × 1	1 × 1 × 1	1.2 × 1.05 × 1.05
DTI acquisition	Axial 2D-EPI	Axial 2D-EPI	Axial 2D-EPI
DTI voxel size (mm)	1.81 × 1.81 × 2	1.98 × 1.98 × 2	0.94 × 0.94 × 2.5
No. of *b* = 0 and b-value (s/mm^2^)	3 × *b* = 0, *b* = 1,000	1 × b = 0, *b* = 1,000	10 × *b* = 0, *b* = 1,000
No. of gradient directions	24	64	2 × 30

*Education levels = a 0–4 score describing degrees/years of education: 0 = high school graduate or above, 1 = some college or above, 2 = associates degree or above, 3 = bachelors or above, 4 = advanced/professional degree (masters or above). Because the PPMI cohort has no collection of education levels, this score was converted from years of education, where 0 = 0–12 years of education, 1 = 13 years of education, 2 = 14–15 years of education, 3 = 16–17 years of education, 4 = 18 and more years of education. Bold: Education levels of all veterans were significantly lower than those of the total healthy controls. For each cohort, we list demographic and multi-center MRI information (sites, scanner manufactures and models, T1WI and DTI acquisition parameters).

### 2.2 Clinical assessments of veterans

All veterans’ multiple chronic symptom conditions were assessed using the centers for disease control and prevention (CDC)/CMI (Fukuda et al., 1998) and Kansas (Steele, 2000) case definition criteria. Under each self-reported symptom category, a positive symptom was defined to have moderate to severe severity that presented for 6 months or more, based on the CDC CFS (chronic fatigue syndrome) Symptom Inventory (Wagner et al., 2005). Clinical diagnosis of PTSD and depression were made according to the DSM-IV-TR Axis I Disorders (First et al., 1997). TBI was diagnosed in accordance with VA and DoD Clinical Practice Guidelines for Management of Concussion and mTBI (Management of Concussion/mTBI Working Group, 2009). Screening for other major symptoms was made using veterans’ self-reports [see detailed descriptions in our previous publication (Zhang et al., 2021)]. The severity of sleep difficulties was assessed using the Pittsburgh Sleep Quality Index (PSQI) (Buysse et al., 1989) global scores (PSQI-glob). Severity of chronic pain was primarily measured by the brief pain inventory (BPI) (Cleeland and Ryan, 1994) short form as a summary score of items 3–6 (BPI-sum). For assessment of chronic symptoms such as sleep and pain, PSQI and BPI are part of the GW common data elements recommendations (Cohen et al., 2022). Additionally, PTSD severity was assessed using the PTSD Checklist for DSM-5 (PCL-5) (Blevins et al., 2015) and depression severity was assessed using the Geriatric Depression Scale (GDS) (Yesavage et al., 1982).

### 2.3 MRI acquisition

Magnetic resonance imaging (MRI) data were acquired at the Palo Alto VA using a 3 Tesla General Electric (GE) Discovery MR750 scanner with an eight channel, GE head coil. The following structural and diffusion MRI sequences were acquired for each subject: (1) 3-dimensional high-resolution T1-weighted image (T1WI) with a spoiled-gradient recalled acquisition (3D-SPGR) in steady state (136 sagittal slices, TR/TE = 7.3/3.0 ms; flip angle = 11°; field of view = 250 mm; slice thickness = 1.2 mm with 0.6 between slices; acquisition matrix = 256 × 256; number of excitations = 1.0; resolution = 1.05 mm × 1.05 mm × 0.60 mm); (2) 57 axial slices of diffusion weighted images (DTI) were acquired using a 2D single-shot EPI sequence with TR/TE = 6600/84.1 ms, 1 mm^3^ × 1 mm^3^ × 2.5 mm^3^ resolution. Ten volumes without diffusion gradients (b0) and 2 sets of scans using 30-directional diffusion weighted gradients (b-value = 1,000 s/mm^2^) were obtained and combined for a 60-directional sequence. MRI acquisition parameters for each of the healthy control cohorts are provided in Table 1.

### 2.4 Structural volume and DTI measures

The T1WI data were processed following standard segmentation and region of interest (ROI) parcellation procedures using FreeSurfer stable version 6.0.0 software package^3^ (Fischl et al., 2002, 2004). Volumetric measures were extracted from prior hypothesized ROIs, including whole brainstem volume, and volume of three brainstem subregions (midbrain, pons, medulla). Volume of the bilateral subcortical ROIs, total cortical and subcortical volumes, and the estimated total intracranial volume (eTIV) were also measured.

To measure the volume of three key brainstem nuclei, including the PAG, LC and RVM, a separate segmentation of the T1WI data was performed using SPM12.^4^ Specifically, individual T1WI were initially segmented to gray matter and white matter maps using SPM12, and further warped to the Montreal Neurological Institute (MNI) standard space using non-linear deformation algorithms in the Advanced Normalization Tools (ANTs) (Avants et al., 2011). A prior atlas of an averaged probabilistic gray matter map and three brainstem nuclei was established in MNI space adopting a thresholding approach on the probabilistic gray matter atlas, which has been described by Brooks and colleagues (Brooks et al., 2017). To calculate volume of the three brainstem nuclei, the binary atlas of the three volume-of-interest (VOI) was inversely transferred from MNI to individual space. The volume (mm^3^) of each brainstem nuclei was measured from the total number of 1 mm^3^ voxels (≥ 50% probability on gray matter maps) that contained in the VOI in subject’s individual space.

Diffusion tensor image (DTI) data were skull-stripped, denoised and distortion corrected using FMRIB’s Diffusion Toolbox within FSL v5.0^5^ (Jenkinson et al., 2012). Subsequent tensor fitting produced maps of diffusion metrics. In individual’s native DTI space, the binary map contains 10 pairs of the major brainstem tracts which were transformed from a brainstem tract atlas in the standardized MNI space, using a recently developed automatic brainstem tractographic approach (Zhang et al., 2020b). Fractional anisotropy (FA) value was extracted bilaterally from the binarized brainstem tracts-of-interest, including medial longitudinal fasciculus (MLF), dorsal longitudinal fasciculus (DLF), superior cerebellar peduncle (SCP), medial forebrain tract (MFT), nigrostriatal tract (NST), corticospinal tract (CST), spinothalamic tract (STT), frontopontine tract (FPT), parietopontine tract (PPT), and temporopontine tract (TPT).

### 2.5 Statistics

All statistics were performed using SPSS (IBM SPSS Statistics for Windows, Version 24.0. Armonk, NY: IBM Corp.). Before statistical analyses, outlying and biasing data points that may have affected analytic results were identified if the absolute standardized Z-score was greater than 3. MRI measures in several regions had fewer than 3 outliers and these individual outliers were removed from all tests. Clinical scores did not have any outliers. A prior linear regression test between clinical and imaging variables revealed a robust relationship between eTIV and the FreeSurfer volumetric measures in all regions, suggesting the necessity to normalize the raw regional volumes (mm^3^) by eTIV to efficiently remove its influence on regional volumetry.

To test MRI differences between deployment subgroups and HC, mean differences of imaging measurement between each group pair were tested as the main fixed effect using a linear mixed effects model. Nested random effects controlled for scanners, as some study cohorts included multiple scanners. Age, gender, education and eTIV were included as covariates in all analyses. Because years of education was missing in one of the control cohorts, we used education levels (0–4 scores describing the highest educational degree) as the education covariate.

In all veterans, each ODS/DS and OEF/OIF subgroup, correlations between each MRI measure and clinical scores (i.e., PSQI-glob, and BPI-sum separately) were tested using Pearson’s partial correlation model, in which the controlling factors included age, sex, education and eTIV. A Fisher’s z score (Diedenhofen and Musch, 2015) was used to compare the statistical differences of imaging-symptom correlations between the two campaign groups. For all tests, a critical significance was set at *p* < 0.01 to adjust for multiple comparisons.

## 3 Results

### 3.1 Demographic and clinical findings

Demographic and clinical information of all 188 veterans and each ODS/DS and OEF/OIF group are shown in Table 2. Out of 188 veterans, 52.1% were ODS/DS veterans with average age of 50.2 years (SD 5.1), 47.9% were OEF/OIF veterans with average age of 39.5 years (SD 9.8). The majority of the ODS/DS (95%) and OEF/OIF (97%) veterans met criteria for CMI. Mann–Whitney U tests showed that the OEF/OIF group had significantly longer combat duration and larger combat exposure scales (*p* < 0.001), marginally more TBI (*p* = 0.01) and PTSD (*p* = 0.02) than the ODS/DS group, whereas the ODS/DS group was significantly older (*p* < 0.001) and had a higher incidence of fibromyalgia (*p* = 0.003) than the OEF/OIF group. Sex, education, CMI, PSQI-glob, and BPI-sum showed no significant differences between deployment subgroups. For detailed demographic information of the age- and gender-matched HC group, see Table 1.

**TABLE 2:** Demographic and clinical data of the veterans’ deployment subgroups.

	OEF/OIF	ODS/DS	All
N.	90	98	188
Age (mean ± SD)	39.5 ± 9.8	**50.2 ± 5.1** ***	45.1 ± 9.4
Age range (years)	23–69	42–67	23–69
Gender N. of M (%)	80 (89%)	88 (90%)	168 (89%)
Handedness N. of right (%)	72 (80%)	79 (81%)	151 (80%)
Ethnicity N. of White (%)	70 (78%)	75 (77%)	145 (77%)
Years of education	14.7 ± 2.4	14.2 ± 2.0	14.4 ± 2.2
Education levels^[a]^ (0| 1| 2| 3| 4)	25| 14| 16| 18| 17	35| 21| 17| 16| 9	60| 35| 33| 34| 26
Combat duration (months)	**18.8 ± 13** ***	11.7 ± 11	15.1 ± 13
Combat exposure scale	**19.5 ± 10** ***	10.4 ± 7.1	14.8 ± 10
**Diagnosis (N.)**			
Meet CMI	87 (97%)	93 (95%)	180 (96%)
Meet Kansas	86 (96%)	93 (95%)	179 (95%)
**Self-reported symptoms (N.)**			
Fatigue/sleep	85 (94%)	91 (93%)	176 (94%)
Pain	85 (94%)	97 (99%)	182 (97%)
Neurological	84 (93%)	91 (93%)	175 (93%)
G.I.	72 (80%)	87 (89%)	159 (85%)
Difficulty breath	65 (72%)	63 (64%)	128 (68%)
Skin	39 (43%)	47 (48%)	86 (46%)
**Positive clinical screens (N.)**			
TBI	**77 (86%)** **	74 (76%)	151 (80%)
PTSD	**77 (86%)** *	70 (71%)	147 (78%)
Depression	59 (66%)	58 (59%)	117 (62%)
Fatigue	50 (56%)	67 (68%)	117 (62%)
Insomnia	71 (79%)	71 (72%)	142 (76%)
OSA	51 (57%)	66 (67%)	117 (62%)
Fibromyalgia	54 (60%)	**78 (80%)** **	132 (70%)
Headache	72 (80%)	71 (72%)	143 (76%)
IBS	60 (67%)	68 (69%)	128 (68%)
SOB	42 (47%)	48 (49%)	90 (48%)
**Clinical scoring**			
Pain scores (BPI-sum)	23.0 ± 7.9	23.0 ± 6.9	23.0 ± 7.4
Sleep scores (PSQI-glob)	13.2 ± 3.8	12.9 ± 4.2	13.0 ± 4.0
PTSD (PCL5)^[b]^	44.6 ± 18.9	41.7 ± 18.8	43.0 ± 18.8
Depression (GDS)^[c]^	8.72 ± 3.3	7.88 ± 3.6	8.29 ± 3.5

^[a]^Education levels = a 0–4 score describing highest degrees of education: 0 = high school graduate or above, 1 = some college or above, 2 = associates degree or above, 3 = bachelors or above, 4 = advanced/professional degree (masters or above).

^[b]^32 are missing.

^[c]^29 are missing. Bold: significantly worsened variables. Significant levels:

*0.01 ≤ *p* < 0.05,

**0.001 ≤ *p* < 0.01,

***p < 0.001. CMI, chronic multisymptom illness; G.I., gastrointestinal system; TBI, traumatic brain injury; PTSD, post-traumatic stress disorder; OSA, obstructive sleep apnea; IBS, irritable bowel syndrome; SOB, shortness of breath; BPI-sum, brief pain inventory sum scores; PSQI-glob, Pittsburgh sleep quality index global scores; PCL5, PTSD checklist 5; GDS, Geriatric depression scale.

### 3.2 Imaging differences between veterans’ groups and healthy controls

**FIGURE 1:**
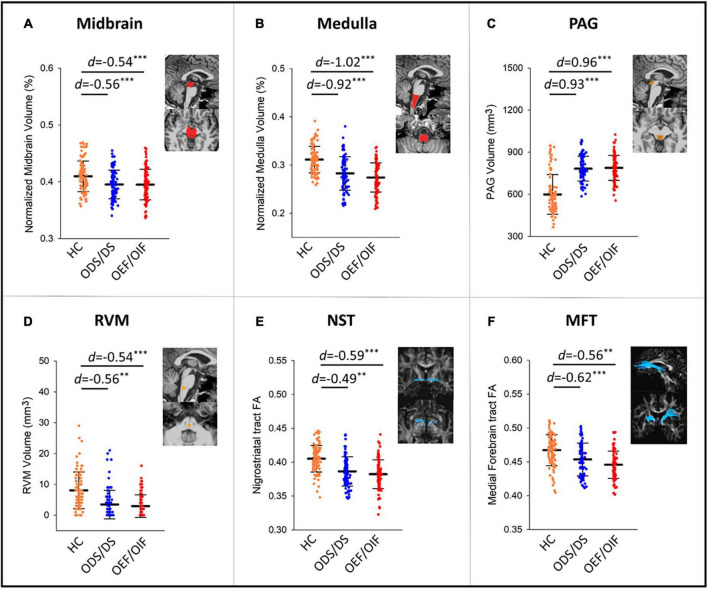
Group comparisons in volume measures in the midbrain **(A)**, medulla **(B)**, PAG **(C)**, RVM **(D)**, and FA measures within NST **(E)**, and MFT **(F)** tracts. PAG, periaqueductal gray; RVM, rostral ventromedial medulla; DLF, dorsal longitudinal fasciculus; NST, nigrostriatal tract; MFT, medial forebrain tract; TPT, temporopontine tract; FA, fractional anisotropy; HC, healthy control; *d*, Cohen’s D effect size. Significant levels: **0.001 ≤ *p* < 0.01, ***p < 0.001.

Figure 1 shows differences of regional volumes between each group pair (i.e., HC vs. OEF/OIF, HC vs. ODS/DS, and OEF/OIF vs. ODS/DS). For volumetric measures, compared to HC, both ODS/DS and OEF/OIF groups showed significantly lower volume in the whole brainstem, including midbrain (OEF/OIF: *d* = −0.56, *p* < 0.001; ODS/DS: *d* = −0.54, *p* < 0.001) and medulla (OEF/OIF: *d* = −0.92, *p* < 0.001; ODS/DS: *d* = −1.02, *p* < 0.001), as well as the RVM (OEF/OIF: *d* = −0.56, *p* = 0.001; ODS/DS: *d* = −0.54, *p* < 0.001). Interestingly, compared to HC, both deployment groups showed significantly greater volume in the PAG (OEF/OIF: *d* = 0.93, *p* < 0.001; ODS/DS: *d* = 0.96, *p* < 0.001). For DTI measures, compared to HC, both ODS/DS and OEF/OIF groups showed significantly reduced FA in the NST (OEF/OIF: *d* = −0.49, *p* = 0.001; ODS/DS: *d* = −0.59, *p* < 0.001), and MFT (OEF/OIF: *d* = −0.62, *p* < 0.001; ODS/DS: *d* = −0.56, *p* = 0.004). Further, compared to HC, OEF/OIF group showed significantly reduced FA in the DLF (*d* = −0.62, *p* = 0.003), SCP (*d* = −0.52, *p* = 0.001) and TPT (OEF/OIF: *d* = −0.68, *p* < 0.001), but FA reduction in the ODS/DS group did not reach a significant level.

Direct comparison between ODS/DS and OEF/OIF showed no significant group differences in any volumetric or DTI measures (see Supplementary Table 1 for detailed results).

### 3.3 Correlates between brainstem MRI, sleep, and pain

**FIGURE 2:**
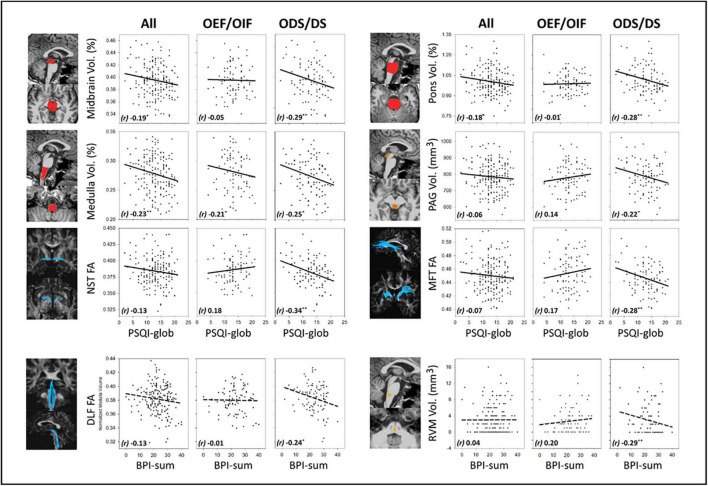
MRI-symptom correlations and between MRI measures (volume in the midbrain, pons, medulla and PAG, FA in the NST and MFT) and PSQI-glob, as well as MRI-symptom correlations between MRI measures (FA in the DLF and volume in the RVM) and BPI-sum, in all veterans, OEF/OIF and ODS/DS veterans, respectively. PAG, periaqueductal gray; NST, nigrostriatal tract; MFT, medial forebrain tract; DLF, dorsal longitudinal fasciculus; RVM, rostral ventromedial medulla; FA, fractional anisotropy; *r*, Pearson’s partial correlation coefficient; BPI-sum, brief pain inventory sum scores, PSQI-glob, Pittsburgh sleep quality index global scores. Solid-line indicates regression of imaging-sleep correlations; dash-line indicates regression of imaging-pain correlations. Significant levels: *0.01 ≤ *p* < 0.05, **0.001 ≤ *p* < 0.01.

Figure 2 shows correlations between MRI measures (volume and FA) and PSQI-glob or BPI-sum, in all veterans, OEF/OIF and ODS/DS subgroup, respectively. Age, sex, education and eTIV were controlled as confounding factors in these partial correlation tests. Supplementary Table 2 lists the full results of imaging-sleep and imaging-pain correlations, after controlling for confounding factors including age, sex, education, eTIV, as well as PTSD and TBI. For sleep, in overall 188 GW veterans, a smaller volume of the brainstem, particularly in the medulla, significantly correlated with increased PSQI-glob (*r* = −0.23, *p* = 0.002 for whole brainstem; *r* = −0.23, *p* = 0.001 for medulla). In the group of 90 OEF/OIF, no significant correlations were found between imaging measures and severities of sleep/pain symptoms; while in the group of 98 ODS/DS, the increased PSQI-glob correlated significantly with smaller brainstem volume (*r* = −0.31, *p* = 0.002 for whole brainstem; *r* = −0.29, *p* = 0.005 for midbrain; *r* = −0.28, *p* = 0.006 for pons), and reduced FA of NST (*r* = −0.34, *p* = 0.001) and MFT (*r* = −0.28, *p* = 0.007). For pain, a significant correlation between smaller volume in RVM (*r* = −0.29, *p* = 0.006) and increased BPI-sum, and a marginal correlation between reduced FA in the DLF (*r* = −0.24, *p* = 0.018) and the increased BPI-sum were observed only in the group of 98 ODS/DS.

### 3.4 Different correlation patterns between OEF/OIF and ODS/DS subgroups

Comparisons between the correlation patterns of the two deployment-based subgroups could reveal if the two campaigns interact with the relationship between MRI and clinical symptoms (i.e., sleep and pain). We found that imaging-sleep and imaging-pain correlations within three anatomical regions showed a significant interaction between OEF/OIF and ODS/DS subgroups. Supplementary Table 2 also lists the results of comparing imaging-symptom correlation patterns between ODS/DS and OEF/OIF subgroups. In particular, correlations between PSQI-glob and FA of the NST (*z* = −3.46, *p* < 0.001), MFT (*z* = −2.99, *p* = 0.001) in the ODS/DS group were significantly different from those imaging-sleep correlations in the OEF/OIF group, stemming from a non-significant relationship in OEF/OIF versus a significant negative relationship in the ODS/DS group. Another significant deployment-based interaction was found in the correlation between BPI-sum and RVM volume (*z* = −3.19, *p* = 0.001), where there is also a non-significant correlation in the OEF/OIF, in comparison with a significant negative correlation in the ODS/DS group.

## 4 Discussion

This is the first study relating structural and diffusion imaging measures to CMI symptoms in a large cohort composed of both Gulf War and Iraq/Afghanistan veterans. Related to our first objective, we found that compared to controls, both ODS/DS and OEF/OIF veterans show smaller volumes in the midbrain, medulla, RVM, larger volume in the PAG, and reduced white matter integrity in the brainstem-spinal cord tracts (i.e., DLF) and brainstem-subcortical tracts (i.e., NST, MLF, and TPT). This in part replicated our previous findings of GWI-related brainstem atrophy that used a different control data set. Regarding our second objective, in the ODS/DS group, we observed significantly negative correlations between increased sleep difficulty and smaller volume in the brainstem, PAG, lower FA in the NST and MFT, whereas no comparable effects were seen in the OEF/OIF group. For the third objective, volumetric and diffusion measures in three ROIs were identified with significantly different imaging-symptom associations between ODS/DS and OEF/OIF groups. All these significant differences were dominated by a significantly negative relationship between increased symptom severity and decreased structural integrity in the ODS/DS group versus no obvious relationship in the OEF/OIF group.

### 4.1 Structural alterations of the brainstem in veterans with CMI

Institute of Medicine (US) Committee on Gulf War and Health (Cory-Slechta and Wedge, 2016) concluded that there is sufficient evidence of an association between deployment to the Gulf War and CMI (including fatigue, musculoskeletal pain, sleep disturbance, cognitive dysfunction, alterations of mood) known as Gulf War Illness (GWI). About 25–35% 1990–91 Gulf War veterans have been estimated to have CMI. Preliminary data suggest that CMI is occurring in veterans of the Iraq/Afghanistan wars as well. Therefore, the present study, potentially for the first time, provides a comparison of both campaigns relating brainstem structures to symptom clusters. According to the existing literature (Christova et al., 2017; Zhang et al., 2020a), CMI manifesting in 1990–91 Gulf War veterans is associated with significantly smaller brainstem volume measured with MRI in comparison with healthy controls. Findings of smaller than normal brainstem volume in the ODS/DS group are largely consistent with our previous observations (Zhang et al., 2020a). More importantly, the current study also observed significantly smaller brainstem volume in CMI from more recent Iraq/Afghanistan conflicts, suggesting that brainstem structural damage is relevant to CMI in veterans from both Middle East campaigns. Interestingly, the smaller volume of the gross brainstem area in both veteran groups was accompanied by a larger-than-HC volume in the PAG that regulates sleep, pain, autonomic activity and stress, together with a smaller-than-HC volume in the RVM that regulates pain perception. The simultaneously larger volume in the PAG and smaller volume in the RVM were both strong findings (Cohen’s effect size: | d| ≥ 0.5), which make this observation unlikely to be accounted for by technical matters such as different MRI scanners and scanning parameters. Additionally, for the first time, we found that compared to controls, the veteran groups exhibited reduced FA in some brainstem-spinal (i.e., DLF, SCP) and brainstem-subcortical (i.e., NST, MLF and TPT) tracts, but not in the brainstem-cortical tracts. Taken together, except the observation of greater PAG volume in veterans, the patterns of smaller volume in the gross brainstem and impaired brainstem tracts are anatomically consistent with the autonomic nervous system that centrally controls body energy, sleep, pain management, and many neurological regulations. Several studies have suggested that dysregulation of the autonomic nervous system is a common feature in GWI (Fox et al., 2019; Martinez-Lavin and Tejada-Ruiz, 2020; Avery et al., 2021). Hence, our findings could suggest that the presence of CMI in veterans deployed to both Gulf War and Iraq/Afghanistan conflicts is highly relevant to structural alterations in the brainstem. Given the fact that sleep difficulty and chronic pain are two major problems in CMI, we discussed the physiological and pathophysiological role of some brainstem nuclei and white matter circuits in regulating sleep and pain functions, and how the brainstem structural changes affect sleep and pain regulation in veterans with CMI in the following sections.

Very few MRI studies have analyzed structural changes of the brainstem nuclei such as PAG, LC, and RVM to detect or differentiate neuropsychological conditions that caused by autonomic dysregulation. Some recent studies have reported volume gain or increased signal intensity of the PAG in episodic migraine (Chen et al., 2017a), medication-overuse headache (Riederer et al., 2012; Chen et al., 2017b), and chronic neuropathic pain- and anxiety-like behaviors (McIlwrath et al., 2020). In the literature, hypoactivation or injury in the RVM has been found in pain patients, and RVM is therefore considered to be engaged in the analgesic process (Cheng et al., 2015; Lerman et al., 2019), while PAG hypertrophies have been suggested to results in dysfunction (decreased inhibitory function) of analgesic systems, and neuronal adaptive changes in response to chronic pain and stressors. Consistent with this notion, the current study found significantly greater volume in the PAG in veterans with CMI. This finding is likely to imply a similar neuronal adaptive change and a structural compensation in response to the complex of chronic pain, sleep difficulties, and neuropsychological symptoms.

Chronic multisymptom illness (CMI) in veterans deployed to both conflicts occur late, usually after returning from deployment. The emergence of CMI may represent a long-term response to numerous distant environmental factors and exposures during deployment. Our findings may possibly indicate a brainstem structural alteration in response to combat exposures, although causality cannot be assumed.

### 4.2 Brainstem nuclei and circuits participate in the dysregulation of sleep/arousal in CMI

The PAG consists of small-sized neurons and largely unmyelinated fibers that interface between the forebrain and brainstem. It is thought to be a center for instinctive behavioral and social reactions that drive emotional experience and physiology in response to threat and injury. The ventrolateral PAG neurons produce neurotransmitters such as glutamate and gamma-amino butyric acid (GABA) that participate in sleep induction and maintenance (Saper and Fuller, 2017). Indeed, GABA receptors are a common target in insomnia treatments (Winsky-Sommerer, 2009). The PAG neurons project fibers to the thalamus, hypothalamus, basal forebrain and cortex which participate in the ascending arousal network (AAN). Specifically, the PAG neurons release active neurotransmitters through their projections to the forebrain, brainstem, and caudal medulla to promote sleep. Dysfunction of these neurons and fiber projections increases wakefulness (Harris, 2005; Saper et al., 2005; Saper and Fuller, 2017). On the other hand, dopaminergic neurons, located in the ventral PAG, may provide the ascending wake-active influence through the projections to ventral tegmental area, striatum and the prefrontal cortex (Lu et al., 2006). PAG subdivisions also activate high general attention, causing hyperarousal symptoms and difficulty in falling sleep (Kozlowska et al., 2015). In other words, long-term, persistent arousal (e.g., insomnia, OSA) may result in structural enhancement in brainstem neurons and networks promoting wakefulness and alertness (e.g., hypothalamus, ventral tegmental area, PAG, and LC). The LC consists of predominantly noradrenergic neurons (Samuels and Szabadi, 2008; Morris et al., 2020). LC releases norepinephrine through fibers that connect to the PAG, tegmentum, raphe nuclei and VTA, subcortical and cerebral cortex, and promote wakefulness, arousal, attention, and concentration (De Cicco et al., 2017). Taken together, the PAG and LC are two major nuclei that are responsible for regulating, particularly promoting arousal, wakefulness, and alertness in the human brain.

Despite both veteran groups having substantially larger PAG volumes than the HC, in this study, a trend of negative correlation (*r* = −0.22, *p* = 0.037; *r* = −0.29, *p* = 0.006 after additionally adjusting for PTSD and TBI) between increased sleep difficulty and PAG volume loss was observed in ODS/DS. As suggested earlier, the increase in PAG volume may be a compensatory or physiological response to the smaller volume of the gross brainstem in the context of CMI symptoms. It is possible to consider that in Gulf War veterans, with increased symptom severity, the predominant change in PAG appeared to be a smaller volume associated dysregulation/decompensation. Nonetheless, we speculate that the change in PAG volume is more likely to be a physiological regulatory response to the gross brainstem injury, rather than a pathogenic factor leading to CMI.

In this study, we found that in ODS/DS, increased sleep difficulty significantly correlated with lower FA in two brainstem circuits (i.e., NST and MFT). As we have discussed in our previous imaging study (Zhang et al., 2021), NST and MFT are involved in the dopaminergic pathways that are responsible for promoting the ascending arousal system. Many investigators have proposed that insomnia is caused by excessive activation of the arousal systems of the brain (Bonnet and Arand, 1997; Nofzinger et al., 2004; Spiegelhalder et al., 2013; Kalmbach et al., 2018), including cell populations and pathways of the cholinergic (e.g., thalamus), serotonergic, dopaminergic (e.g., PAG, ventral tegmental area), and noradrenergic (e.g., LC) systems that project to the hypothalamus and basal frontal cortices (Harris, 2005; Saper et al., 2005; Saper and Fuller, 2017). Yet little is known about the influence of dopaminergic pathways on chronic sleep disorders. Animal studies (Gujar et al., 2011; Mullin et al., 2013) have reported that increases in dopamine activity in the dopaminergic nuclei following sleep deprivation may promote wakefulness to compensate for sleep loss. However, one animal study (Fifel et al., 2018) showed that long-term sleep deprivation resulted in decreased dopamine activity in SN and ventral tegmental area, which is consistent with our findings that chronic sleep difficulties are associated with reduced fiber integrity in the dopaminergic circuits. Further investigations will be needed to understand the mechanisms of changes in dopaminergic tracts under chronic sleep disturbances.

### 4.3 Brainstem nuclei and circuits participate in the dysregulation of pain in CMI

There is increasing evidence suggesting PAG and brainstem circuits participate in the descending pain modulatory pathway. Once the ascending peripheral pain signal reaches the somatosensory cortex, it triggers the descending pain regulation pathways. The descending pain regulation activates the PAG, which releases endogenous opioids and relays to the RVM, and further down to the dorsal horn of the spinal cord through stimulations of the descending anti-nociceptive pathways (e.g., DLF tract) to block the ascending painful signals (Heinricher et al., 2009). Deep brain stimulation of the PAG has been suggested to release endogenous opioids for relief of pain (Sims-Williams et al., 2017). An extensive number of functional MRI studies in various pain conditions and heat-stimulated pain have reported hyperactivation of the PAG and LC, supporting the analgesic responses of the PAG—LC—RVM axis (Napadow et al., 2019).

Hyperactivation of the PAG-RVM axis has been posited to represent an analgesic response to pain stimuli in healthy individuals (Tracey et al., 2002; Mills et al., 2018). With the exception of previously reported hypertrophy of the PAG in the context of chronic headache, structural changes regarding PAG-RVM axis in chronic musculoskeletal pain and fibromyalgia, commonly seen in Gulf War veterans, has to our knowledge never been reported before. Previous structural MRI studies, however, have consistently reported that other chronic pain states are associated with smaller gray matter volume (Zhang et al., 2018) and lower fiber integrity (Geisler et al., 2020; Zhang et al., 2020b) of the PAG-RVM axis. In this study, we observed a significant correlation between increased pain intensity and decreased RVM volume, and a trend of negative relationship between increased pain intensity and disrupted connection (FA reduction) in the DLF (fiber tract linking hypothalamus, PAG, and RVM) in the ODS/DS veterans. Together with our finding that both ODS/DS and OEF/OIF veterans had smaller RVMs than healthy controls, the damage to the PAG-RVM axis and the potentially resulting disruption of effective endogenous analgesia may play an important role in GW-related chronic pain.

### 4.4 Different structural responses to sleep and pain in the two campaigns

As discussed earlier, external stimuli (e.g., sleep difficulty and chronic pain) drive and trigger the activity of the internal neuro-regulation through the autonomic nervous system within the brainstem. We hypothesized that decreased structural integrity may potentially lead to impaired endogenous regulation. Therefore, the relevance of brainstem structural alterations in the context of sleep and pain problems is an important focus in GWI research. In the ODS/DS veterans, we found that FA of the brainstem-subcortical tracts (including NST and MLF) is significantly lower relative to HC, and further reduced with increased sleep difficulty. In the ODS/DS veterans, we also observed that volume of the RVM is significantly smaller relative to HC, and further reduced with increased pain difficulty. These findings largely replicated our previous reports (Golier et al., 2007; Zhang et al., 2020a), suggesting that brainstem damage may be a key feature of GWI (i.e., ODS/DS veterans who present CMI). However, these symptom-related brainstem alterations appeared to be different in the OEF/OIF group. Although OEF/OIF veterans also showed smaller brainstem gross volume and lower brainstem tract integrity compared with HC, no significant correlation was found between these brainstem structures and sleep difficulty or pain intensity in this group. These findings suggested different physiopathologic mechanisms underlying ODS/DS and OEF/OIF veterans although both groups presented similar CMI. One major concern leading to these observations might be the different combat-time and age between the two groups. Combat/environmental exposures, uses of anti-nerve gas agents and multiple vaccinations could also be influencing factors. Further investigations are worthwhile to reveal the biological basis of the different imaging-symptom relationships in Gulf War and Iraq/Afghanistan veterans.

### 4.5 Limitations

The current study lacks a healthy veteran’s control group. Healthy civilians from public data sources were used instead. Thus, the findings from group comparisons could potentially reflect a limited characterization of GWI, which is thought to be a post-deployment illness due to exposures to particular neurotoxic circumstances. Also, there is no comprehensive clinical information available for the HC data. This could limit how specific the present study could be in examining the influences due to these conditions. A second limitation is that the observation of simultaneously larger PAG volumes and smaller RVM volumes could not preclude the possibility of MRI technical issues (e.g., low-resolution, and measurement variations induced by different cohorts/scanners/parameters). The current results need to be replicated with other unrelated data. For volumetry of the brainstem small nuclei, improvements of using high-field high-resolution MRI (Priovoulos et al., 2018) provide the possibility of enhanced accuracy that might detect nuanced findings consistent with previous autopsy results. Another limitation is that the study only focused on investigating sleep and pain related brainstem alterations. Besides sleep and pain, chronic fatigue, pulmonary, gastrointestinal problems and neurological conditions (e.g., headache, impaired memory, and concentration) may have a significant impact on brainstem structures. Assessment of cognitive batteries is included in our ongoing study strategies to reveal its impact on the brainstem in CMI/GWI. Further investigations of the relationship between brainstem structures and other symptom domains (e.g., fatigue, autonomic, neurocognitive status) in GW veterans will be needed.

## 5 Conclusion

The present study showed evidence of smaller brainstem volume and lower white matter integrity of the brainstem-spinal and brainstem-subcortical tracts in veterans deployed to both Gulf War and Iraq/Afghanistan conflicts. This study also showed that substantially smaller brainstem volume and lower integrity of the dopaminergic and pain-control fiber tracts are associated with increased sleep disturbance and pain intensity in 1990–91 Persian Gulf War veterans, but not in Iraq/Afghanistan veterans. Although it is unclear whether brainstem structural alterations constitute the primary pathogenesis of GWI, evidence from our and other groups seem to point to this area as a crucial part of CMI pathology. These findings provide important implications for future clinical care and treatment development.

## Data availability statement

The original contributions presented in this study are included in this article/Supplementary material, further inquiries can be directed to the corresponding author.

## Ethics statement

The study involving human subjects was reviewed and approved by the Institutional Review Boards of Stanford University, National Institute of Mental Health, and PPMI participating sites: Baylor College of Medicine, John Hopkins University, Emory University, The Parkinson’s Institute and El Camino Hospital, Northwestern University, Mellen Center Cleavland Clinic, Philipps-University of Marburg, Parkinson’s Disease and Movement Disorders Center of Boca Raton, University of Tuebingen. The studies were conducted in accordance with the local legislation and institutional requirements. The participants provided their written informed consent to participate in this study.

## Author contributions

YZ: Conceptualization, Data curation, Formal analysis, Methodology, Resources, Visualization, Writing – original draft. MM: Data curation, Methodology, Resources, Writing – review & editing. JJ: Investigation, Resources, Writing – review & editing. JC: Investigation, Resources, Writing – review & editing. PB: Investigation, Project administration, Writing – review & editing. JA: Funding acquisition, Investigation, Resources, Writing – review & editing. AF: Conceptualization, Funding acquisition, Investigation, Project administration, Resources, Writing – review & editing.

## Footnotes

1
https://nda.nih.gov

2
https://ida.loni.usc.edu/

3
http://surfer.nmr.mgh.harvard.edu/

4
https://www.fil.ion.ucl.ac.uk/spm/software/spm12/

5
http://www.fmrib.ox.ac.uk/fsl
