# Supplementary material for: The role of the brainstem in sleep disturbances and chronic pain of Gulf War and Iraq/Afghanistan veterans
Source: Front Mol Neurosci. 2024 Jan 8;16:1266408. doi: 10.3389/fnmol.2023.1266408 (PMC10800562; doi:10.3389/fnmol.2023.1266408)
Supplement: Supplementary file 1 [file Table_1.docx]

**Supplementary Material**

**Supplementary Table 1.** Mean and standard deviation (SD) of MRI measures in healthy control (HC) and veteran subgroups. Group differences were estimated as fixed effects based on linear-mixed model, adjusted by age, gender, education and eTIV.

| **Regions** | **HC** | **OEF/OIF** | **ODS/DS** | **HC vs. OEF/OIF** | | **HC vs. ODS/DS** | | **ODS/DS vs. OEF/OIF** | |
| --- | --- | --- | --- | --- | --- | --- | --- | --- | --- |
|  | Mean (SD) | Mean (SD) | Mean (SD) | Cohen's -*d* | *p*-value | Cohen's -*d* | *p*-value | Cohen's -*d* | *p*-value |
| **Normalized Volume (%)** | | | | | | | | | |
| Total Cortices | 30.79 (1.96) | 31.96 (1.78) | 30.96 (1.75) | 0.149 | *n.s.* | 0.056 | *n.s.* | -0.007 | *n.s.* |
| Total Subcortices | 3.700 (0.239) | 3.828 (0.271) | 3.759 (0.209) | 0.165 | *n.s.* | 0.139 | *n.s.* | 0.053 | *n.s.* |
| Cerebellum | 3.612 (0.349) | 3.625 (0.324) | 3.647 (0.347) | -0.227 | *n.s.* | 0.103 | *n.s.* | 0.266 | *n.s.* |
| Thalamus | 0.466 (0.039) | 0.479 (0.041) | 0.461 (0.036) | 0.073 | *n.s.* | -0.082 | *n.s.* | -0.163 | *n.s.* |
| Caudate | 0.228 (0.024) | 0.229 (0.023) | 0.222 (0.022) | -0.070 | *n.s.* | -0.301 | 0.040 | -0.110 | *n.s.* |
| Putamen | 0.315 (0.033) | 0.323 (0.036) | 0.318 (0.031) | -0.010 | *n.s.* | 0.149 | *n.s.* | 0.223 | *n.s.* |
| Pallidum | 0.128 (0.012) | 0.125 (0.012) | 0.127 (0.015) | -0.308 | 0.040 | -0.123 | *n.s.* | 0.055 | *n.s.* |
| Hippocampus | 0.263 (0.028) | 0.279 (0.023) | 0.277 (0.021) | 0.357 | 0.019 | 0.189 | *n.s.* | 0.096 | *n.s.* |
| Amygdala | 0.109 (0.010) | 0.111 (0.012) | 0.113 (0.012) | 0.169 | *n.s.* | 0.169 | *n.s.* | 0.179 | *n.s.* |
| Whole Brainstem | 1.744 (0.148) | 1.686 (0.126) | 1.705 (0.142) | **-0.473** | **0.002** | -0.375 | 0.042 | 0.096 | *n.s.* |
| Midbrain | 0.409 (0.028) | 0.395 (0.025) | 0.395 (0.027) | **-0.560** | **<0.001** | **-0.540** | **<0.001** | -0.018 | *n.s.* |
| Pons | 1.006 (0.100) | 0.989 (0.078) | 1.017 (0.102) | -0.275 | *n.s.* | -0.102 | *n.s.* | 0.192 | *n.s.* |
| Medulla | 0.311 (0.028) | 0.283 (0.035) | 0.274 (0.030) | **-0.921** | **<0.001** | **-1.015** | **<0.001** | -0.070 | *n.s.* |
| **Volume (mm^3^)** | | | | | | | | | |
| PAG | 603.7 (148.4) | 782.6 (88.9) | 788.0 (89.4) | **0.925** | **<0.001** | **0.958** | **<0.001** | 0.115 | *n.s.* |
| LC | 166.7 (80.7) | 157.7 (54.9) | 170.4 (56.2) | -0.274 | *n.s.* | 0.134 | *n.s.* | 0.105 | *n.s.* |
| RVM | 7.842 (5.55) | 3.478 (4.62) | 2.938 (3.64) | **-0.558** | **0.001** | **-0.535** | **<0.001** | -0.035 | *n.s.* |
| **Fractional Anisotropy (FA)** | | | | | | | | | |
| DLF | 0.394 (0.022) | 0.379 (0.020) | 0.383 (0.020) | **-0.616** | **0.003** | -0.214 | *n.s.* | 0.234 | *n.s.* |
| MLF | 0.499 (0.038) | 0.478 (0.026) | 0.490 (0.025) | -0.232 | *n.s.* | -0.068 | *n.s.* | 0.270 | *n.s.* |
| SCP | 0.411 (0.023) | 0.396 (0.022) | 0.404 (0.022) | **-0.518** | **0.001** | -0.284 | *n.s.* | 0.173 | *n.s.* |
| NST | 0.405 (0.020) | 0.386 (0.022) | 0.382 (0.021) | **-0.494** | **0.001** | **-0.592** | **<0.001** | 0.229 | *n.s.* |
| MFT | 0.467 (0.023) | 0.454 (0.026) | 0.447 (0.022) | **-0.622** | **<0.001** | **-0.555** | **0.004** | 0.073 | *n.s.* |
| CST | 0.538 (0.025) | 0.537 (0.020) | 0.532 (0.018) | 0.189 | *n.s.* | 0.046 | *n.s.* | 0.023 | *n.s.* |
| STT | 0.497 (0.025) | 0.493 (0.018) | 0.491 (0.017) | 0.102 | *n.s.* | 0.015 | *n.s.* | -0.041 | *n.s.* |
| FPT | 0.483 (0.019) | 0.474 (0.023) | 0.469 (0.018) | -0.180 | *n.s.* | -0.510 | 0.040 | -0.004 | *n.s.* |
| PPT | 0.532 (0.027) | 0.524 (0.019) | 0.519 (0.019) | -0.179 | *n.s.* | -0.281 | *n.s.* | -0.064 | *n.s.* |
| TPT | 0.448 (0.023) | 0.431 (0.020) | 0.430 (0.020) | **-0.677** | **<0.001** | -0.357 | 0.019 | 0.304 | 0.042 |

Abbreviations: PAG = periaqueductal gray, LC = locus coeruleus, RVM = rostral ventromedial medulla, DLF = dorsal longitudinal fasciculus, MLF = medial longitudinal fasciculus, SCP = superior cerebellar peduncle, NST = nigrostriatal tract, MFT = medial forebrain tract, CST = corticospinal tract, STT = spinothalamic tract, FPT = frontopontine tract, PPT = praietopontine tract, TPT = temporopontine tract.

**Bold:** significantly smaller variables (*p* < 0.01)

**Supplementary Table 2.** Partial correlation between MRI measures and PSQI-glob / BPI-sum scores controlling for age, sex, education, eTIV, PTSD, and TBI.

| **Correlations** | **Regions** | **Total** | | **Deployment-Subgroups** | | | | | |
| --- | --- | --- | --- | --- | --- | --- | --- | --- | --- |
|  |  | **All veterans (n=188)** | | **OEF/OIF**  **(n=90)** | | **ODS/DS**  **(n=98)** | | **ODS/DS *vs.* OEF/OIF** | |
|  |  | Coefficient | *p*-value | Coefficient | *p*-value | Coefficient | *p*-value | *Test statistic z* | *p*-value |
| PSQI-glob × Volumes | Total Cortices | 0.017 | *n.s.* | -0.070 | *n.s.* | 0.070 | *n.s.* | 0.945 | *n.s.* |
|  | Total Subcortices | -0.079 | *n.s.* | 0.082 | *n.s.* | -0.217 | 0.043 | -2.034 | 0.021 |
|  | Cerebellum | -0.162 | 0.034 | -0.135 | *n.s.* | -0.180 | *n.s.* | -0.310 | *n.s.* |
|  | Thalamus | -0.097 | *n.s.* | 0.002 | *n.s.* | -0.200 | *n.s.* | -1.380 | *n.s.* |
|  | Caudate | -0.051 | *n.s.* | 0.056 | *n.s.* | -0.113 | *n.s.* | -1.139 | *n.s.* |
|  | Putamen | 0.111 | *n.s.* | 0.145 | *n.s.* | 0.092 | *n.s.* | -0.360 | *n.s.* |
|  | Pallidum | -0.110 | *n.s.* | 0.064 | *n.s.* | -0.264 | 0.012 | -2.254 | 0.012 |
|  | Hippocampus | -0.146 | *n.s.* | -0.122 | *n.s.* | -0.166 | *n.s.* | -0.300 | *n.s.* |
|  | Amygdala | -0.062 | *n.s.* | 0.067 | *n.s.* | -0.144 | *n.s.* | -1.426 | *n.s.* |
|  | Whole Brainstem | **-0.239** | **0.002** | -0.005 | *n.s.* | **-0.396** | **<0.001** | -2.789 | **0.003** |
|  | Midbrain | **-0.201** | **0.008** | -0.024 | *n.s.* | **-0.336** | **0.001** | -2.187 | 0.014 |
|  | Pons | **-0.213** | **0.005** | 0.065 | *n.s.* | **-0.383** | **<0.001** | -2.281 | 0.011 |
|  | Medulla | **-0.199** | **0.009** | -0.146 | *n.s.* | -0.254 | 0.025 | -0.759 | *n.s.* |
|  | PAG (mm^3^) | -0.096 | *n.s.* | 0.119 | *n.s.* | **-0.288** | **0.006** | **-2.796** | **0.003** |
|  | LC (mm^3^) | -0.054 | *n.s.* | -0.098 | *n.s.* | -0.032 | *n.s.* | 0.445 | *n.s.* |
|  | RVM (mm^3^) | -0.104 | *n.s.* | -0.098 | *n.s.* | -0.220 | 0.038 | -0.840 | *n.s.* |
| PSQI-glob × | DLF | -0.139 | *n.s.* | -0.078 | *n.s.* | -0.210 | 0.048 | -0.907 | *n.s.* |
| FA | MLF | 0.059 | *n.s.* | 0.002 | *n.s.* | 0.083 | *n.s.* | 0.547 | *n.s.* |
|  | SCP | -0.174 | 0.021 | -0.209 | *n.s.* | -0.190 | *n.s.* | 0.133 | *n.s.* |
|  | NST | -0.137 | *n.s.* | 0.147 | *n.s.* | **-0.362** | **<0.001** | **-3.542** | **<0.001** |
|  | MFT | -0.058 | *n.s.* | 0.167 | *n.s.* | **-0.296** | **0.005** | **-3.192** | **0.001** |
|  | CST | 0.004 | *n.s.* | 0.022 | *n.s.* | -0.035 | *n.s.* | -0.384 | *n.s.* |
|  | STT | -0.043 | *n.s.* | -0.012 | *n.s.* | -0.086 | *n.s.* | -0.499 | *n.s.* |
|  | FPT | -0.033 | *n.s.* | 0.111 | *n.s.* | -0.200 | *n.s.* | -2.097 | 0.017 |
|  | PPT | -0.108 | *n.s.* | -0.048 | *n.s.* | -0.165 | *n.s.* | -0.796 | *n.s.* |
|  | TPT | -0.132 | *n.s.* | -0.136 | *n.s.* | -0.123 | *n.s.* | 0.089 | *n.s.* |
| BPI-sum × Volumes | Total Cortices | -0.148 | *n.s.* | -0.121 | *n.s.* | -0.125 | *n.s.* | -0.027 | *n.s.* |
|  | Total Subcortices | -0.063 | *n.s.* | -0.015 | *n.s.* | -0.036 | *n.s.* | -0.141 | *n.s.* |
|  | Cerebellum | -0.149 | *n.s.* | -0.136 | *n.s.* | -0.100 | *n.s.* | 0.245 | *n.s.* |
|  | Thalamus | -0.020 | *n.s.* | -0.022 | *n.s.* | 0.044 | *n.s.* | 0.445 | *n.s.* |
|  | Caudate | -0.099 | *n.s.* | -0.074 | *n.s.* | -0.163 | *n.s.* | -0.607 | *n.s.* |
|  | Putamen | 0.025 | *n.s.* | 0.000 | *n.s.* | 0.093 | *n.s.* | 0.625 | *n.s.* |
|  | Pallidum | 0.014 | *n.s.* | 0.073 | *n.s.* | 0.004 | *n.s.* | -0.466 | *n.s.* |
|  | Hippocampus | -0.104 | *n.s.* | -0.087 | *n.s.* | -0.055 | *n.s.* | 0.215 | *n.s.* |
|  | Amygdala | -0.017 | *n.s.* | 0.063 | *n.s.* | -0.055 | *n.s.* | -0.794 | *n.s.* |
|  | Whole Brainstem | -0.091 | *n.s.* | 0.069 | *n.s.* | -0.163 | *n.s.* | -1.574 | *n.s.* |
|  | Midbrain | -0.071 | *n.s.* | 0.115 | *n.s.* | -0.178 | *n.s.* | -1.985 | 0.024 |
|  | Pons | -0.074 | *n.s.* | 0.087 | *n.s.* | -0.134 | *n.s.* | -1.496 | *n.s.* |
|  | Medulla | -0.096 | *n.s.* | -0.057 | *n.s.* | -0.125 | *n.s.* | -0.462 | *n.s.* |
|  | PAG (mm^3^) | 0.005 | *n.s.* | 0.059 | *n.s.* | -0.058 | *n.s.* | -0.787 | *n.s.* |
|  | LC (mm^3^) | 0.075 | *n.s.* | 0.099 | *n.s.* | 0.059 | *n.s.* | -0.270 | *n.s.* |
|  | RVM (mm^3^) | 0.046 | *n.s.* | 0.180 | *n.s.* | **-0.280** | **0.008** | **-3.149** | **0.001** |
| BPI-sum × | DLF | -0.153 | 0.045 | -0.059 | *n.s.* | -0.233 | 0.028 | -1.198 | *n.s.* |
| FA | MLF | 0.038 | *n.s.* | 0.078 | *n.s.* | -0.050 | *n.s.* | -0.864 | *n.s.* |
|  | SCP | -0.108 | *n.s.* | -0.018 | *n.s.* | -0.198 | *n.s.* | -1.227 | *n.s.* |
|  | NST | -0.129 | *n.s.* | -0.041 | *n.s.* | -0.162 | *n.s.* | -0.822 | *n.s.* |
|  | MFT | -0.001 | *n.s.* | 0.084 | *n.s.* | -0.059 | *n.s.* | -0.965 | *n.s.* |
|  | CST | 0.082 | *n.s.* | 0.075 | *n.s.* | 0.118 | *n.s.* | 0.293 | *n.s.* |
|  | STT | 0.050 | *n.s.* | 0.089 | *n.s.* | 0.065 | *n.s.* | -0.162 | *n.s.* |
|  | FPT | 0.028 | *n.s.* | 0.154 | *n.s.* | -0.058 | *n.s.* | -1.437 | *n.s.* |
|  | PPT | 0.075 | *n.s.* | 0.070 | *n.s.* | 0.126 | *n.s.* | 0.380 | *n.s.* |
|  | TPT | -0.004 | *n.s.* | -0.036 | *n.s.* | 0.085 | *n.s.* | 0.817 | *n.s.* |

Abbreviations: PAG = periaqueductal gray, LC = locus coeruleus, RVM = rostral ventromedial medulla, DLF = dorsal longitudinal fasciculus, MLF = medial longitudinal fasciculus, SCP = superior cerebellar peduncle, NST = nigrostriatal tract, MFT = medial forebrain tract, CST = corticospinal tract, STT = spinothalamic tract, FPT = frontopontine tract, PPT = praietopontine tract, TPT = temporopontine tract, FA = fractional anisotropy, BPI-sum = brief pain inventory sum scores, PSQI-glob = Pittsburgh sleep quality index global scores, *r* = Pearson’s partial correlation coefficient, *z* = Fisher’s Z score for comparison of correlations from two different samples.

**Bold**: Critical level of significance: *p* < 0.01
